# The Promise of Novel Molecular Markers in Bladder Cancer

**DOI:** 10.3390/ijms151223897

**Published:** 2014-12-22

**Authors:** Jahan Miremami, Natasha Kyprianou

**Affiliations:** Departments of Urology and Molecular Biochemistry, University of Kentucky College of Medicine, Lexington, KY 40536, USA; E-Mail: jd.miremami@uky.edu

**Keywords:** bladder cancer, miRNA, tumor markers, urinary markers, urothelial carcinoma

## Abstract

Bladder cancer is the fourth most common malignancy in the US and is associated with the highest cost per patient. A high likelihood of recurrence, mandating stringent surveillance protocols, has made the development of urinary markers a focus of intense pursuit with the hope of decreasing the burden this disease places on patients and the healthcare system. To date, routine use of markers is not recommended for screening or diagnosis. Interests include the development of a single urinary marker that can be used in place of or as an adjunct to current screening and surveillance techniques, as well identifying a molecular signature for an individual’s disease that can help predict progression, prognosis, and potential therapeutic response. Markers have shown potential value in improving diagnostic accuracy when used as an adjunct to current modalities, risk-stratification of patients that could aid the clinician in determining aggressiveness of surveillance, and allowing for a decrease in invasive surveillance procedures. This review discusses the current understanding of emerging biomarkers, including miRNAs, gene signatures and detection of circulating tumor cells in the blood, and their potential clinical value in bladder cancer diagnosis, as prognostic indicators, and surveillance tools, as well as limitations to their incorporation into medical practice.

## 1. Introduction

### 1.1. Burden and Challenge

In 2013 there were an estimated 72,570 cases of and 15,210 deaths associated with bladder cancer of urothelial origin, making it the fourth most common cancer and having the fifth highest cancer incidence in the US [1]. Relatively long survival times and a 50%–70% recurrence rate mandates aggressive surveillance that contributes to bladder cancer incurring the highest cost per patient ($96,000–$187,000 at 2001 levels) [2,3] and a total cost to the healthcare system greater than $3.4 billion per annum [4,5]. This poses a significant burden to patients, families, and the healthcare system. A large portion of this burden stems from stringent surveillance protocols used to detect recurrence of disease. Current surveillance after treatment usually consists of urine cytology and cystoscopy every three months for the first three years, every six months for the next one to three years, and annually thereafter. Close surveillance is required because roughly 50%–70% of NMIBC cases (70% of total cases at first presentation), consisting of Ta stage, Tis stage, and T1 stage disease, will recur within the first two years after transurethral resection [2].

Bladder cancer is often diagnosed by cystoscopy with urine cytology as an adjunct during the workup of hematuria. Cystoscopy is invasive and has a high sensitivity, except in cases of Carcinoma *in situ*, which may be grossly difficult to detect [6]. Cystoscopy requires experience on the part of the operator for thorough inspection and accurate diagnosis. Urine cytology has a high specificity (90%–96%) but lacks sensitivity, especially in low-grade disease [7]. These factors necessitate the development of relatively non-invasive, cost-effective tests with equivalent or improved sensitivity and specificity compared to the current tools. The ultimate goal of these novel tests would be to aid in the risk-stratification of patients, serve as prognostic indicators for individual patients, determining the molecular signature of an individual’s disease to predict therapeutic response and to be used as a cheap, non-invasive surveillance tool that is simple to interpret to decrease the need for frequent surveillance cystoscopy, ultimately decreasing patient burden and costs to the healthcare system.

### 1.2. Bladder Cancer Biomarkers in Clinical Use

Numerous potential markers have been described and are under investigation, though relatively few have been validated to be clinically useful. The FDA has approved BTA (Bladder Tumor Antigen) stat^®^, BTA TRAK^®^, NMP22 (Nuclear matrix protein)/BladderChek^®^ and UroVysion™ for diagnosis and follow-up, while ImmunoCyt™/uCyt™ is approved for follow-up. Other promising, previously described, markers include BLCA-4, CYFRA 21-1, Survivin, UBC™, and DD23. Among these, only Urovysion^®^ has become a frequently used tool because it has been shown to be more sensitive than urinary cytology by detecting aneuploidy in chromosomes 3, 7, 17 and 9p21 through FISH analysis. A meta-analysis of 14 studies comparing FISH to urine cytology showed a pooled sensitivity/specificity of 72%/83% and 42%/96%, respectively. However, this sensitivity advantage of FISH over urine cytology was nearly eliminated when superficial (Ta) disease was excluded from the analysis, showing the utility of FISH with the diagnosis of Ta stage disease compared to urine cytology [8]. Widespread incorporation of FISH into clinical practice has been limited due to its expense and inability to replicate the high specificities of urine cytology, where a positive result is especially helpful for ruling in the diagnosis of bladder cancer.

The BTA stat^®^/BTA TRAK^®^, detecting Human Complement Factor H, assays are also FDA approved for diagnosis and follow-up. BTA stat^®^ is a bedside point-of-care immunochromatographic assay, while BTA TRAK^®^ is a quantitative ELISA assay. Both have comparable ranges of sensitivity/specificity of roughly (53%–83%)/(67%–72%) for BTA stat^®^ and (66%–72%)/(51%–75%) for BTA TRAK^®^ [2], but the former costing only a fraction of the latter. A major disadvantage of these assays, as well as other protein-based assays, is that false-positive results can occur with certain changes of the urinary milieu, such as highly concentrated urine, cystitis, hematuria, presence of indwelling instrumentation/stents, and previous treatment with BCG.

NMP22 (Nuclear matrix protein) is ubiquitous protein that has been studied for possible role as a marker for bladder cancer with assays detecting the release of the protein from apoptotic cells. This assay also has the disadvantage of false positive results in the setting of benign inflammatory conditions, like those stated in the above paragraph, though it can be used after treatment with BCG. The prospective UroScreen trial investigated the use of NMP22 in the screening of 1772 high-risk individuals with exposure to aromatic amines, with resulting 224 positive test results correctly identifying only 6 cases of bladder cancer. The resulting sensitivity/specificity/negative predictive value/positive predictive value of the test was accordingly 97%/28%/99%/12%. These findings argued strongly against a recommendation for NMP22 for screening of high risk populations because of a high false positive rate in benign inflammatory conditions and the low prevalence of disease, even in high risk populations [9].

Individual markers have not shown sufficient diagnostic power to replace current strategies, presumably due to the release of proteins from apoptotic cells in benign conditions, contributing to the false positive results, thereby decreasing specificity of these markers [10]. Additionally, the relatively rapid rate of discovery of potential markers has resulted in confusion of which markers or combinations to use in certain clinical situations and has limited the determination of cost-effectiveness and further validation of tumor markers [11]. Combining tests may result in improved accuracy at the expense of conducting multiple tests and adding costs. At present, there is lack of evidence-based guidelines supporting the effective integration of tumor biomarkers into clinical practice due to limitations of validation and increasing costs for routine utility, despite the advances in technology.

## 2. Discussion

### 2.1. Adjuncts to Current Strategies

A strategy being aggressively pursued in recent studies is towards the identification of markers that can be potentially used to improve the sensitivity when combined with urine cytology. A recent study by Todenhoffer *et al.* [12], beginning with 808 patients suspected of having urothelial carcinoma was conducted to investigate the value of combinations of Cytology, uCyt™, FISH, and/or NMP22-ELISA in the diagnosis of bladder cancer (see Table 1). All patients underwent urethrocystoscopy, upper-tract imaging, FISH, uCyt™, and NMP22-ELISA, as well as TURBT if there were positive findings on urethrocystoscopy. Bladder cancer was diagnosed in 115 patients. The best single overall tests were FISH and cytology with an area under the curve of 0.78 and 0.79, respectively. Increased sensitivities and AUC (0.83) were attained when the two tests were combined. AUC was further increased to 0.86 when uCyt™ was added as the third test. Interestingly enough, the NMP22 addition did not further influence the AUC.

A clinical study involving 100 patients (60 with urothelial carcinoma, 20 urological patients without UC, and 20 healthy volunteers) investigated the combination of urinary survivin, measured by ELISA, or urinary hyaluronidase, measured by RT-PCR, with urine cytology. The results indicated an improvement in sensitivity to 83.33% and 90%, respectively, compared to 38.33% of cytology alone. Moreover, approximately 95% sensitivity was achieved when both survivin and hyaluronidase were combined with cytology [13]. The same investigative team conducted a similar study of 66 patients divided into two groups, 20 with a benign urologic condition and 46 with bladder cancer (29 with UC, 17 with SCC) after cystoscopy and histopathology comparing urinary survivin and matrix metalloproteinases (MMP) 2 and 9, detected with gel zymography. The results demonstrated an increased sensitivity from 50% to 84.7%, when either urinary survivin or MMP was combined with cytology; the highest sensitivity was attained at 95.6%, when both survivin and MMP profiles were combined with cytology [14]. This evidence identified the power of the combination value of two biological players in bladder cancer diagnosis.

**Table 1:** Clinical value of novel bladder tumor markers.

Combinations/Novel Markers	Value	Reference
**Cytology ± FISH ± uCyt™ ± NMP22**	Diagnosis	Todenhoffer *et al.* 2013 [13]
**Cytology ± Survivin ± MMP**	Diagnosis	Eissa *et al.* 2013 [15]
**Cytology ± Survivin ± Hyaluronidase**	Diagnosis	Eissa *et al.* 2013 [14]
**Cystoscopy ± NMP22 ± FISH**	Surveillance	Kamat *et al.* 2011 [16]
**Nicotinamide *N*-Methyltransferase**	Diagnosis	Sartini *et al.* 2013 [17]
**P-Cadherin**	Prognosis	Wang *et al.* 2014 [18]
**EpCAM**	Prognosis	Bryan *et al.* 2014 [19]
**miR 130b + 141+ 199-3p + 205**	Diagnosis	Ratert *et al.* 2013 [20]
**miR 141**	Prognosis	Ratert *et al.* 2013 [20]
**miR 205**	Prognosis	Ratert *et al.* 2013 [20]
miR 29c*	Prognosis	Rosenberg *et al.* 2013 [21]
miR 187 + 18a* + 25 + 142-3p + 140-5p + 204	Diagnosis and Prognosis	Mengual *et al.* 2013 [22]

We must also consider the additional role of biomarkers in identifying disease characteristics in the clinical setting and guiding clinical decision-making when considered alongside other disease characteristics, such as tumor stage, grade, size, focality, prior recurrence and presence of CIS, being referred to as a “molecular grade”. One such molecular grade includes two marker mutations, FGFR3 activating mutations present in genetically stable disease and conferring a good prognosis, as well as major alterations in Ki-67 profile, usually associated with a poor prognosis. Patients can then be characterized into molecular grades 1, 2 or 3, and may have prognostic value when added to the European Organization for Research and Treatment of Cancer Risk Scores or other risk stratification tools. In a study by Van Rhijn *et al.* [23], multivariable analysis showed mG3 had independent significance for progression and disease-specific survival while high EORTC risk scores were significant for recurrence and progression. When the molecular grading system was added to the EORTC risk score assessment as a model to predict progression, predictive accuracy increased from 74.9% to 81.7%. This illustrates the value of biomarkers as an indication of disease characteristics and their value when considered alongside other prognostic factors and risk-stratification tools.

### 2.2. Cost-Effectiveness

Bladder cancer contributes significantly to the financial burden of our healthcare system, much of which is incurred by the need for frequent, invasive surveillance procedures due to the propensity for disease recurrence. Bladder cancer biomarkers that can reliably detect disease or indicate propensity for recurrence would aid the clinician in identifying patients that require less frequent cystoscopy and cytology, decreasing costs associated with surveillance. Kamat *et al.*, conducted a prospective trial set out to evaluate how NMP22/BladderChek^®^ and Urovysion^®^ would affect overall sensitivity and costs when added to cystoscopy when screening for recurrent bladder cancer in 200 consecutive patients who had previously treated Ta, T1 or CIS disease [16]. The five strategies compared were cystoscopy alone, cystoscopy and NMP22, cystoscopy and FISH, cystoscopy and cytology, and cystoscopy and positive NMP22 confirmed by positive FISH with detection rates of 52%, 56%, 72%, 60% and 56%, respectively. Costs per tumor identified were increased substantially with the addition of the bladder tumor markers, and their addition was associated with a high false positive rate. Costs per tumor detected for each group were $7692, $12,000, $26,462, $11,846 and $10,292, respectively. Despite somewhat better detection with the addition of relatively cheap urinary marker tests, the associated high false-positive rate leads to unnecessary, expensive, and invasive procedures.

## 3. Value and Validation

With technological advances allowing the analysis of biological markers and increasing interest in urinary markers for bladder cancer, a plethora of candidates have been identified and will be identified in the future. Sartini *et al.* [17], identified Nicotinamide *N*-methyltransferase as being highly expressed in bladder cancer after cDNA macroarray of tumor and normal appearing tissue in the same patient. Tissue samples were obtained from 28 patients after radical cystectomy and urine samples were obtained from separate cohorts of 26 patients with high-grade UC and 16 healthy volunteers. cDNA macroarray, RT-PCR, western blot analysis, and enzyme catalytic activity assay conducted on samples found NNMT mRNA overexpression and markedly increased enzyme activity in 100% of bladder cancer compared to adjacent normal tissue while having higher enzyme levels in urine samples from the confirmed bladder cancer group compared to the healthy volunteers. There is tremendous promise in the value of this enzyme to serve as a potential diagnostic tool that calls for validation of the early findings in a large patient cohort.

Immnohistochemical profiling of P-cadherin expression, a calcium dependent transmembrane glycoprotein molecule, functionally assigned the critical role of a mediator of cell-cell adhesion interactions, in 110 tumor specimens from bladder cancer patients after TURBT, revealed that 54 patients exhibited high expression, while 56 had relatively low expression of P-cadherin [18]. The clinical follow-up was for a median of 35 months; during this period 43 patients recurred and 25 progressed to advanced disease. Statistical evaluation revealed a significant correlation between high P-cadherin expression levels with disease progression and between low expression with progression-free survival in this patient cohort (*p* = 0.034). This evidence implicated high expression of P-cadherin with progression of NMIBC, but external validation and prospective evaluation are required to determine the clinical value as a prognostic indicator that may ultimately allow for the stratification of patients.

More recently a study by Bryan *et al.* [19], interrogated the expression pattern of epithelial cell adhesion molecule (EpCAM), using ELISA, in urine of 607 patients with initial cystoscopic findings suggestive of primary bladder cancer compared to 53 non-cancer controls to determine its utility in diagnosis and risk stratification of patients. Urinary EpCAM was significantly elevated in UC in comparison to healthy controls (median 6.74 pg per mg of creatinine *vs.* 3.86, *p* = 0.0025); However, this increase was confined to high grade and late-stage disease. The findings revealed that elevated urinary EpCAM is specific for grade 3 or greater disease and T2+ disease. Moreover, there was a strong association with a significant (1.8-fold) increase in UC-specific death. This potential marker begs for exhaustive validation to define its clinical value in detecting advanced, aggressive disease.

Identification of novel markers is an ongoing pursuit and continued investigative efforts have taught us that non-invasive urinary tumor markers are not limited to use in detection of disease. Certain markers and marker combinations can be used to predict progression and aid in the classification of an individual’s disease. This can, in turn, help guide the clinician in the determination of aggressiveness of treatment and surveillance by risk-stratifying individual patients. A wealth of potential biomarkers has been discovered due to technological innovations in their identification but because these technologies tend to be costly, they tend to be applied to relatively small sample sizes, affecting their statistical power. Further investigation in longitudinal studies would ideally be conducted in large cohorts that are disease-free initially and subject to long-term follow-up, but since the incidence of disease is relatively low and large cohorts are costly, these studies tend to be conducted in at-risk populations. Few of the findings in observational studies progress to be validated in large-cohort, randomized clinical studies to clearly determine how their use affects patient outcomes and costs [11]. This is crucial step in establishing the clinical value of a biomarker in bench-to-bedside transition.

## 4. miRNA—The Emerging Promise in Bladder Cancer Prognosis

A rapidly expanding area within the scope of urinary bladder tumor markers is the identification of miRNAs and their emerging role in the regulation of gene expression. miRNAs are non-coding RNAs that prevent the translation of mRNAs with their complement sequences into proteins. [24] The resultant effect depends upon the function of the protein that is coded by the silenced mRNA. This new molecular dynamic in control of gene expression can ultimately lead to aberrant expression of genes that may predispose normal cells to malignant transformation. Dysregulated miRNAs in UC may serve as screening tests, prognostic indicators, and aid in the identification of novel targets for therapy when used alone or as combination. Elegant molecular studies by Ratert *et al.* [20], interrogated fifteen miRNA candidates after screening of a total of 723 miRNAs for potential diagnostic and prognostic value, based on 8 non-malignant and 16 malignant bladder cancer tissue specimens with further validation with RT-(q)PCR of the 15. Seven miRNAs (miR-20a, miR-106b, miR-130b, miR-141, miR-200a, miR-200a* and miR-205) were up-regulated and eight (miR-100, miR-125b, miR-130a, miR-139-5p, miR-145*, miR-199a-3p, miR-214 and miR-222) were found to be down-regulated. The group included four previously described (miR-141, miR-199a-3p, miR-205 and miR-214) markers differentially expressed in NMIBC *vs.* MIBC. Significantly enough, ROC analysis revealed high area under curve values for all 15 miRNAs (AUC > 0.8), indicating a solid ability to distinguish between normal tissue and UC. The combination of four miRNAs (miR-130b, miR-141, miR-199-3p and miR-205) resulted in correct classification of 100% of tissue samples. Moreover, Kaplan-Meier analyses revealed significant differences in the overall survival for miR-141 (log-rank test = 5.427; *p* = 0.02) and miR-205 (log-rank test = 4.114; *p* = 0.04). Separate Kaplan-Meier analyses for NMIBC and MIBC identified a prognostic potential for miR-141 only in MIBC (log-rank test = 3.144; *p* = 0.08), but not NMIBC. This initial translational evidence however created a rigorous platform implicating a potential value for miRNAs as diagnostic and possibly prognostic markers in bladder cancer (the limitations of the retrospective nature of the study notwithstanding).

More recent studies by Rosenberg *et al.* [21], pursued a possible miRNA indicator that could predict progression of disease by comparing miRNA profiles of NMIBC that did not progress and those that later progressed. Specifically miR-29c*, a miRNA species involved in down-regulating DNA methyltransferases as well as up-regulating demethylating genes, was severely decreased in tumors that progressed; Significantly enough it showed potential value to risk-stratify patients with T1 disease into those with high *vs.* low risk of progression. Out of the 36 cases harboring high expression (>9.71) of this miRNA, only two total cases progressed, only one of which was in the first five years. Low expression (<8.71) group had a median progression-free survival of 35 months, 50% of cases later progressed, and miR-29c* has been shown to have significant value when risk stratifying NMIBC (*p* < 0.0001).

A molecular signature of miRNA combinations provides a dynamic platform for establishing strong diagnostic value and predicting aggressiveness of UC. Mengual *et al.* [22], found that out of 22 miRNAs tested, three miRNAs (miR-187, miR18a*, and miR-25) were over-expressed and three (miR-142-3p, miR-140-5p and miR-204) were under-expressed in UC compared to healthy controls. Internal validation of this 6-miRNA diagnosis model allowed the accurate diagnosis of UC in patients with a mean sensitivity, specificity, and AUC of 83.74 ± 0.057, 87.64 ± 0.043 and 0.91 ± 0.024, respectively. In addition, overexpression of miR-92a and underexpression of miR-125b could differentiate high-grade UC from low-grade with a mean sensitivity, specificity, and AUC of 82.7 ± 0.076, 81.3 ± 0.102 and 0.822 ± 0.061, respectively. Fuelled by the research momentum surrounding the discovery of the miRNA functions, one recognizes that the clinical value and applications of such miRNA signatures, require external validation of the initial small-scale studies in large randomized trials, as well as comparisons against the current standards of care.

## 5. Microarray Gene Expression Profiling

Gene signatures have emerged with high promise as powerful molecular tools enabling the differentiation between stages of disease and precise identification of those with aggressive characteristics that may eventually develop into invasive forms. A prospective, multicenter study by Descotes *et al.* that included 108 bladder tumors utilized microarray gene expression profiles to identify particular molecular signatures that represent certain pathological stages or grades [25]. The signatures of 976 probes was able to correctly identify 92.8% (26/28) of muscle-invasive (≥T2) disease and 66.3% (53/80) of NMIBC. Out of the NMIBC, 86.7% of papillary Ta and 68.8% of T1a tumors were correctly classified. Interestingly, T1b tumor signatures were more akin to those in the muscle invasive categories, 84.2% (16/19) of T1b tumors were classified in the muscle invasive “gene clusters”. Molecular signatures have been shown to have the ability to characterize a patient’s disease based upon the stage of disease and potentially identify a subset of disease that may progress to invasive forms.

## 6. Circulating Tumor Cells—“New Kids on the Block”

A large majority of efforts in bladder cancer biomarker discovery and validation has been focused upon analysis of urine because of its intimate contact with the primary tumor and the non-invasive nature of a urine-based screening test. More recently however and with the application of advanced state-of-the art technology, the focus has been creatively shifted on the detection and analysis of circulating tumor cells (CTCs) in patients’ blood. CTCs may act as a “liquid biopsy” or act as an indication that an individual’s disease is more likely to recur/progress. Their potential value has been pursued with rigor and anticipation in multiple human malignancies, including breast and prostate cancer [26,27]. Incorporation of this tool into a clinician’s armamentarium may allow for the extrapolation of the risk for disease recurrence/progression in an individual patient. The clinician could then tailor the aggressiveness of therapy and surveillance procedures in individual patients.

In pursuit of the potential prognostic value of CTCs detection in patients with NMIBC, Gazzaniga *et al.* [28], demonstrated that their presence (CTCs) was associated with a significantly shorter time to recurrence of disease. CTCs were detected in eight out of their 44 patients with NMIBC and had an average time to first recurrence of 6.5 *vs.* 21.7 months in the patients where CTCs were not detected (*p* < 0.001). Furthermore, there was an association between CTCs positive patients with a designation of T1 disease (*n* = 26), whereas CTCs were not detected in any of the Ta disease (*n* = 18). Overall, seven out of the eight CTCs harboring patients had local recurrence and progressed to MIBC in the 24-month follow up while only 13 out of 36 of the CTC negative patients had local recurrence, none of which progressed to MIBC. The findings suggest that it is possible to identify a subset of patients who may merit more aggressive therapy and surveillance to prevent recurrence, and possible progression to MIBC. Median time to progression was not reached due to the short 24-month follow-up. Further analysis at the end of five years of follow-up is anticipated despite the limitations of the small sample size in the study.

A separate study by Flaig *et al.* [29], embarked to determine the incidence and prognostic value of CTCs in UC. CTCs were detected in five of 30 subjects (range 1–6, only 1 having > 5 CTCs per 7.5 mL blood sample) with clinically localized disease and seven of 14 with metastatic disease (range 1–177, 5 had > 5 CTCs per 7.5 mL blood sample). The presence of CTCs in patients with metastatic disease was associated with poor survival (<1 year), with a median survival of 156 *vs.* 337 days in patients lacking CTCs. There was no difference of survival between the CTC positive and CTC negative patients with localized disease after two years. These findings suggest that the detection of CTCs have a potential prognostic value in patients with advanced disease, though analyses of larger cohorts are required to determine a meaningful “positive” result, as opposed to the simple presence or absence of CTCs, and what that means for the patient. CTCs represent an additional potential tool to aid decision-making in the treatment of patients with bladder cancer. As technology advances in refining analysis of this aggressive subset of cells, improved insights into the prognosis and therapeutic response are expected.

## 7. Conclusions

The discovery and validation of tumor biomarkers provides much promise but the field remains in its infancy. Many markers have shown potential as diagnostic tools, screening tools, and prognostic indicators with the ultimate goal of improving existing care of bladder cancer, decreasing costs associated with stringent surveillance protocols, and decreasing patient discomfort. Though no single marker has been able to outperform current modalities due to disease heterogeneity and high false-positive rates, they have been shown to improve diagnostic accuracy when used as an adjunct to current strategies or when multiple markers are used. Interrogation of miRNA species as potential tumor markers due to their critical involvement in gene regulation has met with promise, especially when miRNAs are collectively profiled into a molecular signature. While controversy still surrounds a potential success of the value of such mRNA signatures in the diagnosis of bladder cancer, one may consider that they may also be predictive of prognosis and differentiating between NMIBC and MIBC. In a personalized medicine approach, risk-stratifying individuals based upon their disease’s molecular signature could be classified by the clinician for therapeutic and surveillance aggressiveness. Investigation into the classification of disease based upon its molecular signature to indicate risk of disease progression and possible therapeutic response has shown promise to one-day guide treatment of an individual’s disease. CTCs have shown considerable prognostic value and serve as an indication in potential therapeutic response in multiple malignancies. The evidence discussed above points to a potentially high prognostic value of CTCs in human bladder cancer progression with the ability to identify a subset of the patient population that may require more aggressive treatment and follow-up to prevent recurrence and/or metastatic progression of the disease. These findings await validation by performance of large randomized trials subject to longer follow-up to increase their impact in a clinical setting of bladder cancer. The International Consultation on Urological Diseases released their recommendations in 2012, stating that tumor marker research is promising but remains investigational [30].

Further validation of markers in randomized, multicenter clinical trials, as well as the conduction of international forums to determine protocols for marker use, are required to establish their clinical utility and support their incorporation and accessibility in the healthcare system. Studies demonstrating the impact of biomarker incorporation into clinical practice on diagnosis and surveillance within distinct patient populations, accounting for race, gender, and socioeconomic status, are required as a novel biomarker test may become expensive due to costs accrued during extensive validation studies.

## Abbreviation

FDA: Food and Drug Administration
NMP22: nuclear matrix protein-22
FISH: fluorescence *in situ* hybridization
ELISA: enzyme linked immunosorbent assay
AUC: area under the curve
TURBT: *trans*-urethral resection of bladder tumor
UC: urothelial carcinoma
SCC: squamous cell carcinoma
MMP: matrix metalloproteinase
NNMT: nicotinamide *N*-methyltransferase
NMIBC: non-muscle-invasive bladder cancer
miRNA: micro ribonucleic acid
EpCAM: epithelial cell adhesion molecule
RT-(q)PCR: real-time quantitative polymerase chain reaction
ROC: receiver operating characteristics
CTC: circulating tumor cells
BCG: bacillus calmette-guerin
CIS: carcinoma *in situ*
EORTC: European Organization for Research and Treatment of Cancer
FGFR3: fibroblast growth factor receptor 3
